# Combined Curvature and Wall Shear Stress Analysis of Abdominal Aortic Aneurysm: An Analysis of Rupture Risk Factors

**DOI:** 10.1007/s00270-022-03140-z

**Published:** 2022-04-12

**Authors:** Biyun Teng, Zhijun Zhou, Yu Zhao, Zhe Wang

**Affiliations:** Department of Vascular Surgery, The First Affiliated Hospital of Chongqing Medical University, No. 1, Youyi Road, Yuanjiagang, Yuzhong District, Chongqing, 400016 China

**Keywords:** Abdominal aortic aneurysm, Curvature, Computational fluid dynamics, Wall shear stress

## Abstract

**Purpose:**

To discuss the risk factors for abdominal aortic aneurysm rupture based on geometric and hemodynamic parameters.

**Methods:**

We retrospectively reviewed the clinical data of those who were diagnosed with an abdominal aortic aneurysm by computed tomography angiography at our hospital between October 2019 and December 2020. Thirty-five patients were included in the ruptured group (13 patients) and the unruptured group (22 patients). We analyzed the differences and correlations of anatomical factors and hemodynamic parameters between the two groups using computational fluid dynamics based on computed tomography angiography.

**Results:**

There were significant differences in the maximum diameter [(79.847 ± 10.067) mm vs. (52.320 ± 14.682) mm, *P* < 0.001], curvature [(0.139 ± 0.050) vs. 0.080 (0.123 − 0.068), *P* = 0.021], and wall shear stress at the site of maximal blood flow impact [0.549(0.839 − 0.492) Pa vs. (1.378 ± 0.255) Pa, *P* < 0.001] between the ruptured and unruptured groups, respectively. And in the ruptured group, wall shear stress at the rupture site was significantly different from that at the site of maximal blood flow impact [0.025 (0.049 − 0.018) Pa vs. 0.549 (0.839 − 0.492) Pa, *P* = 0.001]. Then, the maximum diameter and curvature were associated with rupture (maximum diameter: OR: 1.095, *P* = 0.003; curvature: OR: 1.142E + 10, *P* = 0.012). Most importantly, curvature is negatively correlated with wall shear stress (*r* = − 0.366, *P* = 0.033).

**Conclusions:**

Both curvature and wall shear stress can evaluate the rupture risk of aneurysm. Also, curvature can be used as the geometric substitution of wall shear stress.

## Introduction

An abdominal aortic aneurysm (AAA) is a permanent, localized aneurysmal dilation with a mortality rate of 65–85% after rupture [1, 2]. Although the standard of AAA surgical intervention is based on the maximum diameter, the prediction of AAA rupture by aneurysm diameter alone has some limitations. It cannot solve AAAs that rupture below a threshold or reach a larger diameter without rupture, nor can it explain the phenomenon that female AAAs are more likely to rupture at smaller sizes.

To explain the above problems, different tools were developed to assess the risk factors of AAA rupture. Computer models are able to individualize aorta of a patient with aneurysm, simulating tissue stress and hemodynamics in the aneurysm. Among them, computational fluid dynamics (CFD) plays an important role to prediction of AAA rupture, and the parameters peak wall stress (PWS) and wall shear stress (WSS) are the indicators in usual. The researchers found PWS to be a significant independent predictor in predicting AAA rupture [3]. WSS, the tangential force exerted on the wall of blood flow, is an important hemodynamic factor regulating the artery. Low WSS may be more accurate than maximum diameter in predicting ruptured AAA [4]. Numerous studies have shown that in AAA, rupture sites occur in areas of low WSS and in most cases are close to areas of flow stagnation with higher intraluminal thrombus deposition [5–8]. In addition, WSS is also used to assess hemodynamics in the patients with thoracic aortic aneurysm and aortic dissection [9, 10].

Undoubtedly, the predictive effect of WSS on AAA rupture is very established. However, CFD models, because of their tools and complex operational procedures, are difficult to construct a hemodynamic model for each AAA patient to predict rupture, and their translation to the clinic has a maximum limit. In addition, the geometric index of AAA is the most fundamental, simple and effective prediction method, which affects the hemodynamics of aneurysm [11]. The latest CFD model showed the effect of AAA neck angle on the distribution of WSS [12]. By constructing six idealized AAA models, the researchers found that the hemodynamic predictor WSS was highly dependent on the shape value (Dmax to height ratio) of AAA [13]. In this study, we aim to evaluate whether AAA geometric parameters (curvature, neck diameter) except the maximum diameter are associated with WSS. The statistically significant correlation index can be used as the geometric substitution of WSS.

## Methods

### Patient Selection

We gathered patients with AAA treated at our hospital between October 2019 and December 2020. AAA patients diagnosed by computed tomography angiography (CTA) at our hospital were included. The exclusion criteria were as follows: suprarenal AAA, traumatic aneurysm, infectious aneurysm, multiple aneurysms, tuberculous aneurysm, and lack of CTA imaging data at our hospital. Patients were divided into ruptured and unruptured groups, as mean as rupture and stable groups.

### Data Collection

Demographic information of AAA patients was collected in our hospital medical record system. And CTA images of AAA patients at admission were saved in our imaging workstation, and we collected CTA images in digital imaging and communications in medicine (DICOM) format. SOMATOM Definition Flash CT Scanners, and all CTA were derived by contrast injection, with contrast agent (Bayer Health Care Co., Ltd) 90 ml, injection speed 4 ml/s, slice thickness was 0.75 mm. Table 1 shows the demographic information of the patients, including sex, age, blood pressure on admission, and previous medical history.

**Table 1:** Demographic characteristics and clinical characteristics of abdominal aortic aneurysm patients

	Ruptured group	Unruptured group	*P* value
	*N* = 13	*N* = 22	
Gender(male/female)	10/3	19/3	0.648
Age(years)	75(78 − 69.5)	71.09 ± 10.38	0.448
Smoking (%)	66.67	68.18	1.000
Drinking (%)	33.33	54.55	0.297
Hypertension (%)	58.33	40.91	0.475
CAD^a^ (%)	16.67	27.27	0.681
Diabetes (%)	8.33	9.09	1.000
SBP^b^ (mmHg)	131.77 ± 39.758	136.95 ± 18.290	0.664
DBP^c^ (mmHg)	80.00(87.50 − 63.50)	80.82 ± 11.065	0.511

^a^*CAD* coronary heart disease

^b^*SBP* systolic blood pressure

^c^*DBP* diastolic blood pressure

### Three-Dimensional Model Construction

**Fig. 1:**
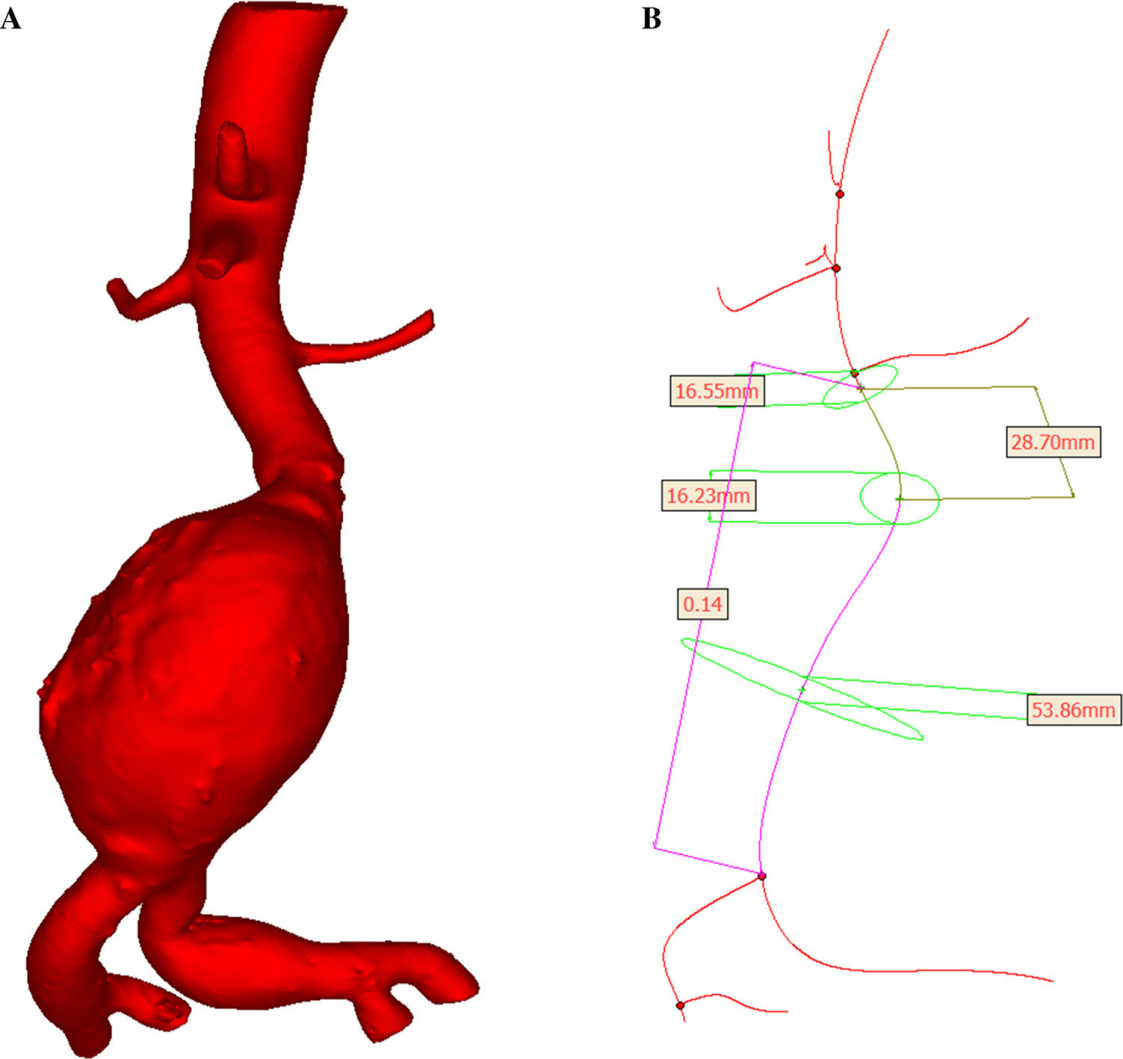
Three-dimensional reconstruction of the abdominal aortic aneurysm (**A**) of one patient, with a maximum diameter of 53.86 mm, neck diameter of 16.23 mm, aneurysm diameter at the level of the lower renal artery of 16.55 mm, neck length of 28.70 mm, and curvature of 0.14 according to centerline measurements (**B**)

We imported CTA images into the reverse engineering software Mimics Medical 20.0 (Materialise, Belgium) for medical image processing. Mimics software is a tool for three-dimensional image generation. Use the threshold tool in Mimics to select the appropriate gray threshold range, display AAA lumen and the related structure. We selected images from 2 cm above the infrarenal aortic aneurysm to the external iliac origin and excluded small branches of the artery (inferior mesenteric artery and renal artery) to decrease the complexity of the geometry. Mimics software reconstructions were generated, and the maximum diameter, neck diameter, and curvature were measured (Fig. 1). The geometric indexes are dependent on the calculation of the cavity center line, which is automatic extraction. Among them, the maximum diameter and neck diameter were calculated perpendicular to the center line; curvature indicates the tortuosity of the AAA, which is the ratio between the straight-line distance and actual distance from the lower renal artery to the bifurcation of the iliac artery. Finally, three-dimensional reconstructions were exported in stereolithography (STL) format.

### Grid Model Construction

**Fig. 2:**
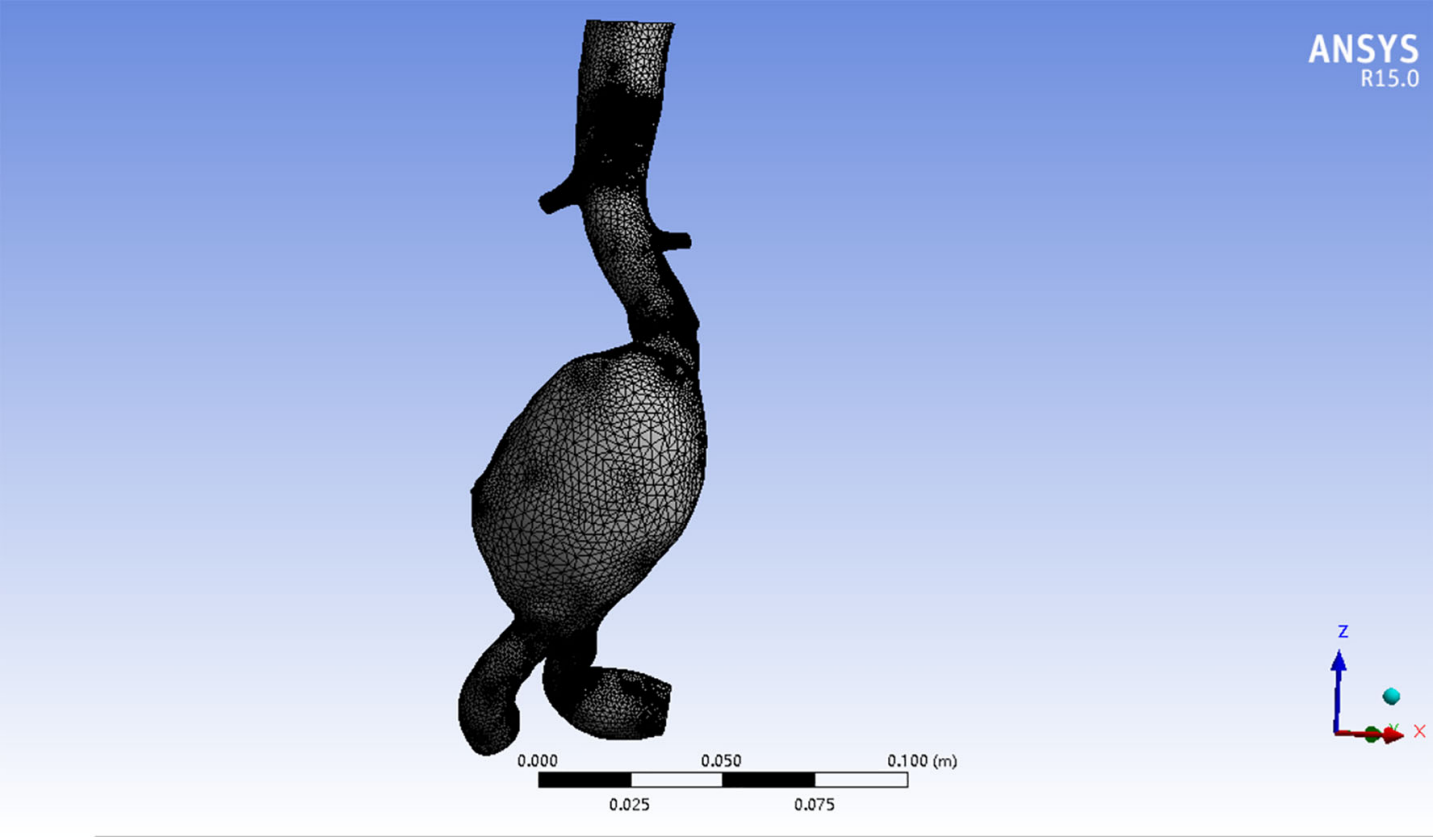
The grid model of abdominal aortic aneurysm. Abdominal aortic aneurysm is divided into several elements by triangular mesh for finite element analysis

Mimics provides a mesh tool named 3-Matic to optimize the three-dimensional geometric model to eliminate small details that do not affect or have less of an impact on the results of the analysis, such as small branch vessels. In 3-Matic, we use denoising processing to improve the accuracy of subsequent analysis and minimize the noise points caused by instruments, blood vessels, environment and human factors during CT scanning. According to the existing point cloud data on AAA model, a grid model is formed based on triangular grid method. In the mesh model, the incompleteness of the CT scan at the edges leads to inevitable gaps and voids, and we use processing such as pasting gaps and filling holes to make the structure of the AAA 3D model more reasonable. The model is made smoother by smoothing mode. After this tool was applied, a three-dimensional hexahedral grid method is also triangle mesh, based on lattice theory was applied for automatic division setting in ANSYS 15.0 (ANSYS, Inc., USA) (Fig. 2).

### Finite Element Analysis

**Fig. 3:**
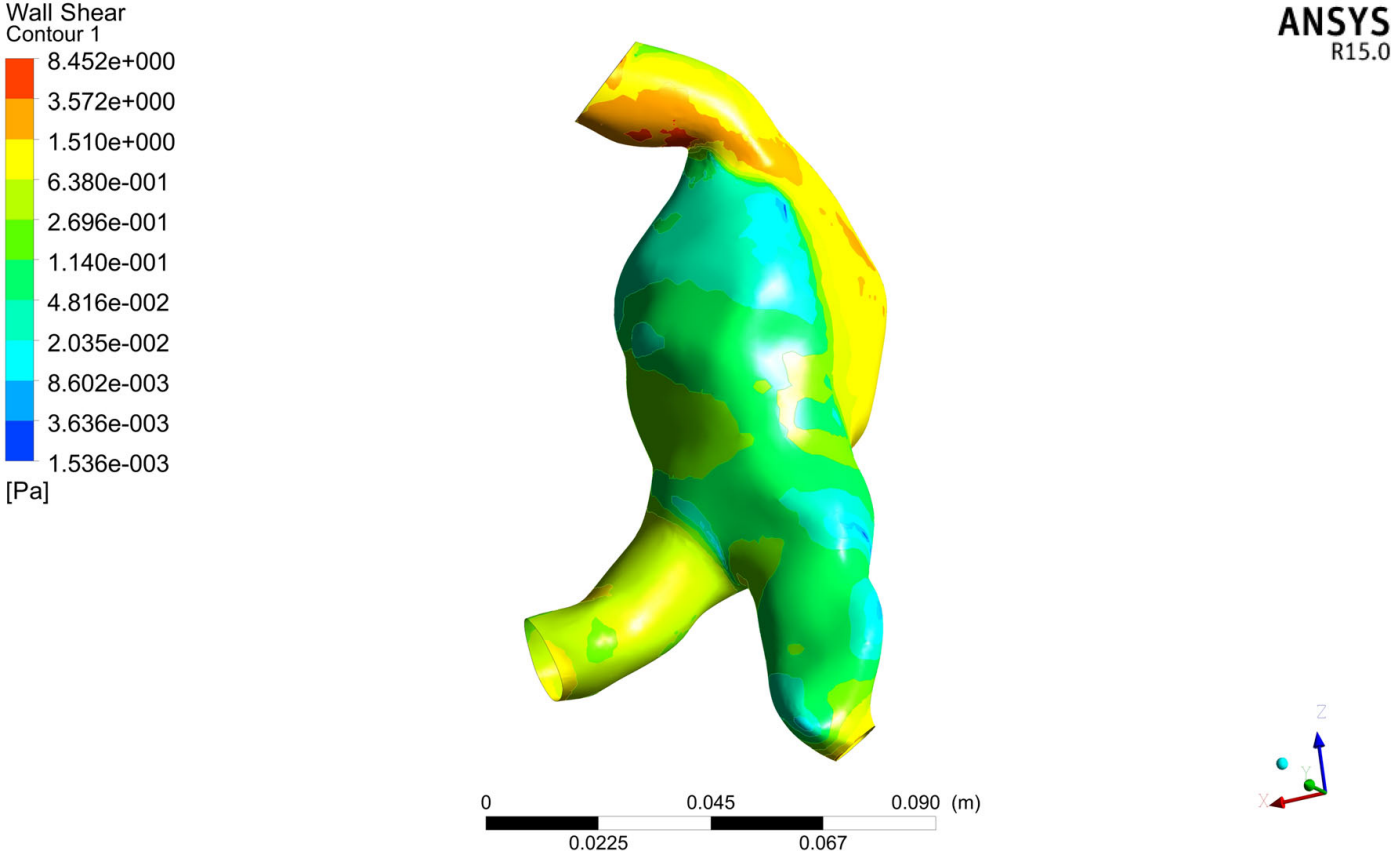
Three-dimensional image of the wall shear stress in an aneurysm. Different colors indicate different levels of stress; red corresponds to high stress, and blue corresponds to low stress

The Navier–Stokes equation and continuity equation of fluid motion under transient conditions were solved by the finite volume method using the ANSYS FLUENT CFD 15.0 solver (ANSYS, Inc., USA). Hemodynamic parameters are time function. One cardiac cycle is set to 0.8 s in human body, and it is set to calculate 5 cardiac cycles totaling 4 s with each step size of 0.01 s, totaling 5 cardiac cycles. Model conditions need to be set during the calculation. Blood was assumed to be a Newtonian and incompressible fluid with a density (*ρ*) of 1.050 g/cm^3^ and a dynamic viscosity (*μ*) of 0.0035 Pa.s [8]. Vascular walls were defined as a nonlinear, isotropic, hyperelastic material with a density, elastic modulus and Poisson's ratio of 2 g/cm^3^, 2.7 MPa, and 0.45, respectively [14]. Then, the inlet velocity was fixed at 0.8 m/s with 140 mmHg of boundary pressure. At the outlet boundary, a zero diffusion flow boundary condition was applied [15]. The wall shear stress (WSS) value and another atlas were then generated by CFD simulation and finite element analysis. The WSS is the tangential force exerted by blood on the vessel wall, and the maximum WSS in the aneurysm was defined as the WSS at the maximum blood flow impact (Fig. 3).

### Statistical Analysis

All statistical analyses were performed using SPSS 26.0 (IBM, the USA). Normally distributed continuous data are presented as the mean ± standard variables, and nonnormal data are presented as the median and interquartile range. Pearson and Spearman correlation analyses were used to evaluate correlations. Comparisons between groups were made using independent *t*-tests and Fisher’s exact tests for normally distributed data and Mann–Whitney *U* tests for nonnormally distributed data. Single factors were analyzed using logistic regression. *P* < 0.05 was considered to indicate a statistically significant difference.

## Results

### Demographic Characteristics

Thirty-five patients were included, with 13 assigned to the ruptured group and 22 to the unruptured group, mostly male, older than 65 years old. In the two groups, the ratio of smokers was greater than 50%. The independent *t*-tests showed that there was no significant difference or no statistical significance between the two groups in gender, age, smoking, drinking hypertension, coronary heart disease (CAD), diabetes and blood pressure, as shown in Table 1.

### Geometric Parameters

**Fig. 4:**
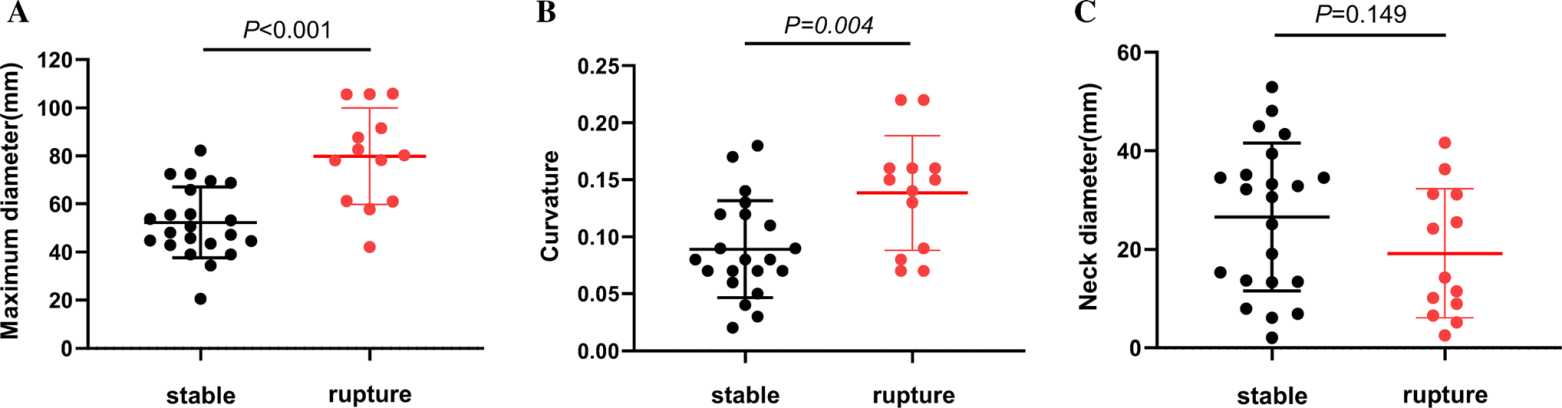
Variation analysis of geometric parameters in two groups. (**A**) The maximum diameter and (**B**) curvature were different in the two groups, *P* < 0.05, but the neck diameter **C** was not statistically different

In 35 cases of AAA, the average maximum diameter of ruptured group was 79.847 ± 10.067 mm, and that of unruptured group (stable group) was 52.320 ± 14.682 mm (Independent *t*-test: *P* < 0.001), indicating that the maximum diameter of ruptured group was significantly larger than that of unruptured group, but it was not limited to the diameter required for surgical intervention in the guidelines. Among them, the maximum diameter of the aneurysm in the rupture group was as low as 42.1 mm, and there was no sign of rupture in the large diameter aneurysm of 80.23 mm (Fig. 4A). Figure 4B shows the difference of curvature between the two groups was statistically significant [0.139 ± 0.050 vs 0.089 (0.10 − 0.070), *P* < 0.05], and the curvature in ruptured group was significantly higher than stable group. However, although the neck diameter is different between rupture and stable groups, it is not statistically significant [(19.148 ± 13.101) mm vs. (26.570 ± 15.030) mm] (Fig. 4C).

We then performed a logistic regression analysis of geometric parameters in 13 patients with ruptured AAA and 22 patients without rupture to conclude that maximum diameter (OR: 1.095, *P* = 0.003) and curvature (OR: 1.142E + 10, *P* = 0.012) were associated with the occurrence of AAA rupture, both as independent risk factors for AAA rupture. In contrast, neck diameter had no association with rupture of AAA (OR: 0.963, *P* = 0.149), shown in Table 2.

**Table 2:** Logistic regression analysis of geometric parameters of abdominal aortic aneurysm

	OR	*P* value	95%CI
Maximum diameter(mm)	1.095	0.003	1.032 − 1.161
Curvature	1.142E + 10	0.012	158.551 − 8.225E + 17
Neck diameter(mm)	0.963	0.149	0.915 − 1.014

### Wall Shear Stress (WSS)

**Fig. 5:**
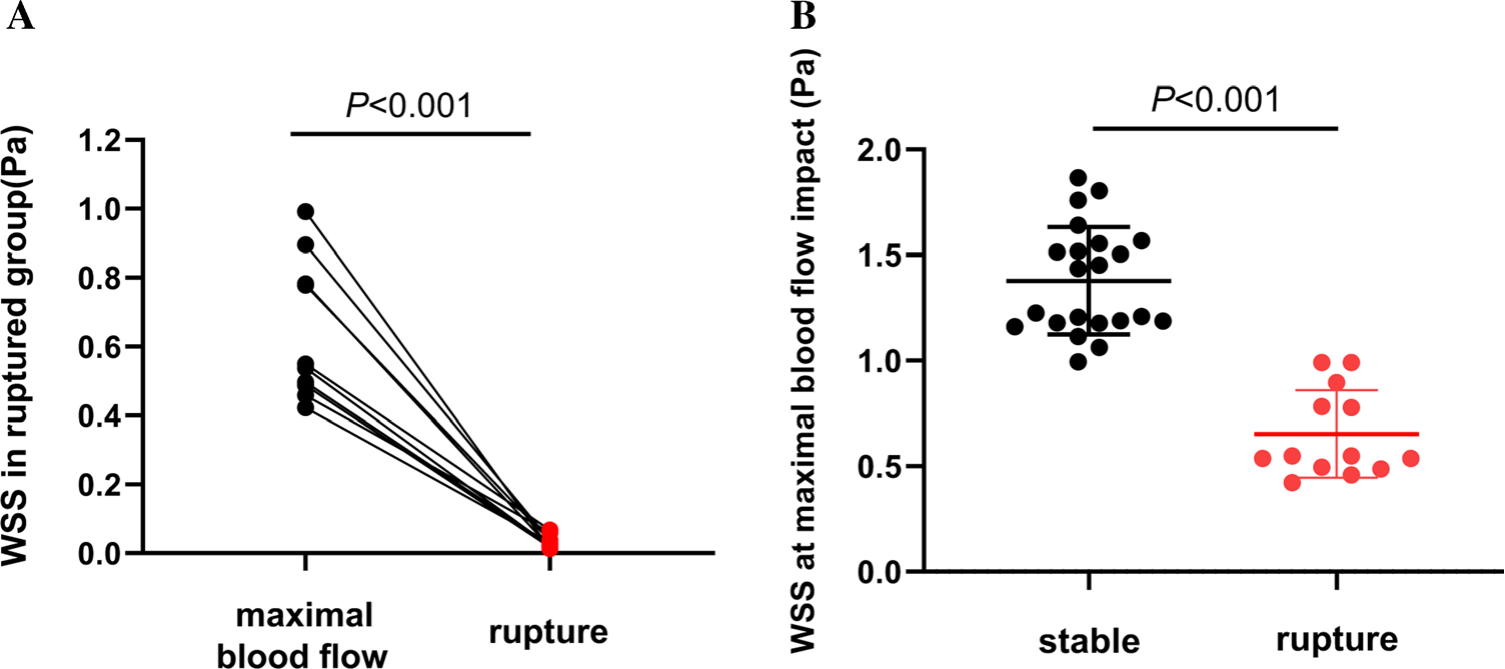
Differences in WSS between ruptured and stable groups. (**A**) In the rupture group, there was a significant difference of WSS between the site of maximal blood flow impact and rupture, with WSS at the rupture site was close to 0. (**B**) Compared with high WSS in the stable group, WSS at the site of maximal blood flow impact was significantly lower in the rupture group

In the ruptured group, the mean WSS at the rupture site was 0.025 (0.049 − 0.018) Pa, which was nearly 20-fold lower than that at the site of maximal blood flow impact, at 0.549 (0.839 − 0.492) Pa. This difference was statistically significant (Wilcoxon signed-rank test, *P* = 0.001), as shown in Fig. 5A. The mean WSS at the site of maximal blood flow impact was 1.378 ± 0.255 Pa in the unruptured group, which was significantly greater than that in the ruptured group (Fig. 5B). These results demonstrate that AAA rupture is more likely to occur in the low WSS region, where blood flow is slow.

### The Correlation Between WSS and Geometric Parameters

**Fig. 6:**
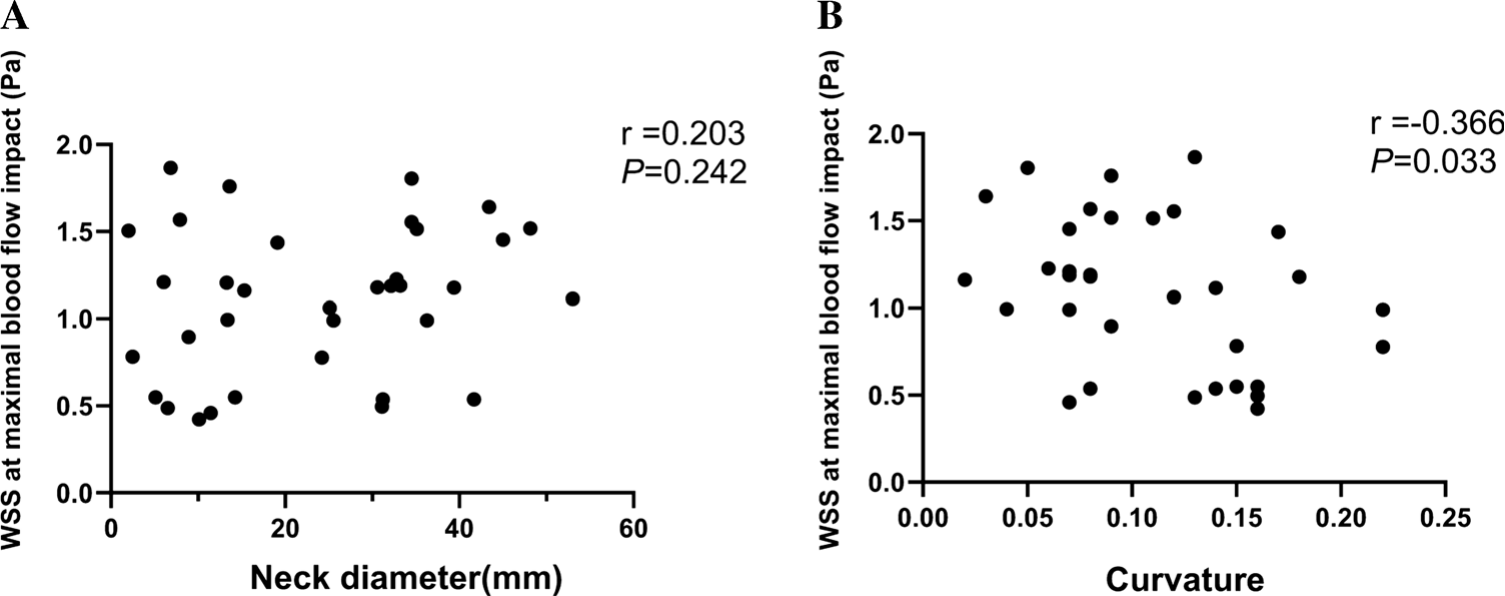
Correlation analysis between WSS at the site of maximal blood flow impact and geometric parameters. (**A**) In 35 patients, we found that neck diameter was not correlated with WSS, while (**B**) curvature was negatively correlated with WSS

In order to find geometric indicators that could replace the hemodynamic parameter WSS, we used correlation analysis to find statistically significant indicators. In 35 patients with AAA, we found there was no statistically significant correlation between neck diameter and WSS at the site of maximal blood flow impact (*r* = 0.203, *P* = 0.242), as shown in Fig. 6A. However, the curvature in Fig. 6B was negatively correlated with the WSS at the site of maximal blood flow impact, and the correlation was statistically significant (*r* = − 0.366, *P* = 0.033).

## Discussion

In recent years, predicting the risk of AAA rupture has been a hot research topic, where model construction and basic research are the main aspects of this research, but have not replaced the treatment strategy of intervening in aneurysms based on the maximum diameter. However, clinicians mostly use CTA, MRI and ultrasound to evaluate AAA in hospitals, and both CFD and 4D flow magnetic resonance imaging (MRI) require mature technical requirements and skilled operational procedures as the basis, which greatly limits the transformation of model construction to the clinic. In this study, we tried to find convenient and effective geometric parameters to replace the most commonly used indicator in model construction-WSS.

Our study analyzed the geometric parameters and WSS in 13 ruptured patients and 22 unruptured patients with AAA. The results showed that the majority of AAA patients were male, with more smoking history, and older than 65 years. Among the AAA geometric parameters, the maximum diameter and curvature in ruptured group were significantly higher than those in unruptured group. And there was no difference in neck diameter between the two groups. Logistic regression analysis shows that the maximum diameter and curvature can predict the rupture of AAA alone, which is an independent risk factor. The maximum diameter, as a criterion for aortic aneurysm repair, is unquestionable in its differential and independent predictive ability. Among them, Fig. 3A shows that large-diameter aneurysms can remain stable, while smaller-diameter AAAs ruptured. In addition, a large number of studies used curvature as an independent indicator in their analyses [16–18]. Recent mouse AAA model studies have shown that mean and Gaussian curvatures are the key geometric parameters for determining rupture, respectively [17].

In our study, consistent with the results of other studies, the decrease in WSS was an essential parameter for AAA rupture, and the WSS at the rupture site was close to 0. Some studies have shown that aortic rupture occurred in areas of low WSS and blood flow recirculation, not areas of high WSS [8]. Exposure to low WSS has resulted in proinflammatory and pro-oxidant expression patterns and decreased aneurysm cell activity [19–21]; additionally, low WSS has been shown to promote intravascular plaque formation and prevent vascular endothelial cells from accessing nutritional blood [22]. These results suggest that the risk of AAA rupture increases with decreasing WSS.

In Fig. 5A, we find that the geometric parameter curvature is negatively correlated with the hemodynamic parameter WSS, and WSS decreases with increasing curvature. The correlation between curvature and WSS emphasizes the importance of geometric shape in evaluating AAA rupture. Curvature can be used as a geometric substitute for WSS except for the maximum diameter to predict AAA rupture. In the correlation analysis of AAA geometry and mechanical parameters, curvature and PWS are common indexes. PWS, as a classical mechanical index, is considered to be significantly higher in ruptured AAA than in intact AAA, especially when approaching rupture [23–25]. It has been hypothesized that enhanced AAA distal surface curvature would lead to greater PWS values; however, there are no results to support this [18]. Another finite element analysis studies the correlation between PWS and AAA geometric parameters in the presence of intracavity thrombosis, indicating that there is no linear relationship between curvature and PWS [26]. However, mean centerline curvature is an important predictor of PWS, with which a high correlation was demonstrated in a model analysis of 39 patients [27]. At present, there are few studies on the correlation between curvature and WSS. We found that as the curvature increases, the WSS becomes lower and will be matched by easier intraluminal thrombus formation, resulting in reduced vessel wall strength and rupture of the AAA.

There are several importation limitations regarding the present study. First, we made several assumptions about the vascular wall, blood flow velocity, blood viscosity, and other factors of the hemodynamic model. These factors may obscure the effect of differences in the vessel wall thickness among individuals and sites, the instability of the blood flow volume and velocity, and the influence of blood viscosity on other factors. Second, currently, research has not been able to establish whether a reduction in WSS is a cause or a consequence of aneurysm rupture. Third, the sample size included is relatively small. Future studies should involve a larger sample size and additional relevant parameters.

## Conclusions

In this study, we analyzed and compared geometric parameters and WSS between the ruptured and unruptured groups and found that maximum diameter, curvature, and WSS were statistically different. The high value of diameter, curvature, and low WSS distinguished patients who were at high risk for rupture. Diameter and curvature can independently predict AAA rupture. We find that curvature is negatively correlated with WSS, which can be used as a geometric substitution of WSS except the maximum diameter.

## Abbreviations

AAA: Abdominal aortic aneurysm
CFD: Computational fluid dynamics
CTA: Computed tomography angiography
WSS: Wall shear stress
PWS: Peak wall stress

## References

[1] Sakalihasan N, Michel J-B, Katsargyris A, Kuivaniemi H, Defraigne J-O, Nchimi A (2018). Abdominal aortic aneurysms. Nat Rev Dis Prim.

[2] Assar AN, Zarins CK (2009). Ruptured abdominal aortic aneurysm: a surgical emergency with many clinical presentations. Postgrad Med J.

[3] Soto B, Vila L, Dilme J, Escudero JR, Bellmunt S, Camacho M (2018). Finite element analysis in symptomatic and asymptomatic abdominal aortic aneurysms for aortic disease risk stratification. Int Angiol.

[4] Qiu Y, Yuan D, Wen J, Fan Y, Zheng T (2018). Numerical identification of the rupture locations in patient-specific abdominal aortic aneurysmsusing hemodynamic parameters. Comput Methods Biomech Biomed Eng.

[5] Doyle BJ, McGloughlin TM, Kavanagh EG, Hoskins PR. From Detection to Rupture: A Serial Computational Fluid Dynamics Case Study of a Rapidly Expanding, Patient-Specific, Ruptured Abdominal Aortic Aneurysm. Computational Biomechanics for Medicine. 2014. p. 53–68.

[6] Zambrano BA, Gharahi H, Lim C, Jaberi FA, Choi J, Lee W (2016). Association of intraluminal thrombus, hemodynamic forces, and abdominal aortic aneurysm expansion using longitudinal CT images. Ann Biomed Eng.

[7] Stevens RR, Grytsan A, Biasetti J, Roy J, Lindquist Liljeqvist M, Gasser TC (2017). Biomechanical changes during abdominal aortic aneurysm growth. PLoS ONE.

[8] Boyd AJ, Kuhn DC, Lozowy RJ, Kulbisky GP (2016). Low wall shear stress predominates at sites of abdominal aortic aneurysm rupture. J Vasc Surg.

[9] Ramaekers M, Adriaans BP, Juffermans JF, van Assen HC, Bekkers S, Scholte A (2021). Characterization of ascending aortic flow in patients with degenerative aneurysms: a 4D flow magnetic resonance study. Invest Radiol.

[10] Peng L, Qiu Y, Yang Z, Yuan D, Dai C, Li D (2019). Patient-specific computational hemodynamic analysis for interrupted aortic arch in an adult: implications for aortic dissection initiation. Sci Rep.

[11] Georgakarakos E, Ioannou C, Papaharilaou Y, Kostas T, Katsamouris A (2011). Computational evaluation of aortic aneurysm rupture risk: What have we learned so far?. J Endovasc Ther Off J Int Soc Endovasc Spec.

[12] Algabri YA, Rookkapan S, Gramigna V, Espino DM, Chatpun S (2019). Computational study on hemodynamic changes in patient-specific proximal neck angulation of abdominal aortic aneurysm with time-varying velocity. Australas Phys Eng Sci Med.

[13] Philip NT, Patnaik BSV, Sudhir BJ (2022). Hemodynamic simulation of abdominal aortic aneurysm on idealised models: investigation of stress parameters during disease progression. Comput Methods Programs Biomed.

[14] Raghavan ML, Vorp DA (2000). Toward a biomechanical tool to evaluate rupture potential of abdominal aortic aneurysm: identification of a finite strain constitutive model and evaluation of its applicability. J Biomech.

[15] Moore JE, Jr., Ku DN. Pulsatile velocity measurements in a model of the human abdominal aorta under resting conditions. J Biomech Eng. 1994;116(3):337–46. 10.1115/1.2895740 7799637

[16] Urrutia J, Roy A, Raut SS, Anton R, Muluk SC, Finol EA (2018). Geometric surrogates of abdominal aortic aneurysm wall mechanics. Med Eng Phys.

[17] Lane BA, Uline MJ, Wang X, Shazly T, Vyavahare NR, Eberth JF (2021). The association between curvature and rupture in a murine model of abdominal aortic aneurysm and dissection. Exp Mech.

[18] Azar D, Ohadi D, Rachev A, Eberth JF, Uline MJ, Shazly T (2018). Mechanical and geometrical determinants of wall stress in abdominal aortic aneurysms: a computational study. PLoS ONE.

[19] Wasserman SM, Mehraban F, Komuves LG, Yang RB, Tomlinson JE, Zhang Y (2002). Gene expression profile of human endothelial cells exposed to sustained fluid shear stress. Physiol Genomics.

[20] Meng H, Tutino VM, Xiang J, Siddiqui A (2014). High WSS or low WSS? Complex interactions of hemodynamics with intracranial aneurysm initiation, growth, and rupture: toward a unifying hypothesis. AJNR Am J Neuroradiol.

[21] Lu G, Huang L, Zhang XL, Wang SZ, Hong Y, Hu Z (2011). Influence of hemodynamic factors on rupture of intracranial aneurysms: patient-specific 3D mirror aneurysms model computational fluid dynamics simulation. AJNR Am J Neuroradiol.

[22] Chang SHU, Tun WANG (2012). Characteristics of hemodynamics in abdominal aortic aneurysm and its treatment. Chin J Pract Surg.

[23] Heng MS, Fagan MJ, Collier JW, Desai G, McCollum PT, Chetter IC (2008). Peak wall stress measurement in elective and acute abdominal aortic aneurysms. J Vasc Surg.

[24] Truijers M, Pol J, Schultzekool L, Van Sterkenburg S, Fillinger M, Blankensteijn J (2007). Wall stress analysis in small asymptomatic, symptomatic and ruptured abdominal aortic aneurysms. Eur J Vasc Endovasc Surg.

[25] Geest JPV, Schmidt DE, Sacks MS, Vorp DA (2008). The effects of anisotropy on the stress analyses of patient-specific abdominal aortic aneurysms. Ann Biomed Eng.

[26] Georgakarakos E, Ioannou CV, Kamarianakis Y, Papaharilaou Y, Kostas T, Manousaki E (2010). The role of geometric parameters in the prediction of abdominal aortic aneurysm wall stress. Eur J Vasc Endovasc Surg.

[27] Giannoglou G, Giannakoulas G, Soulis J, Chatzizisis Y, Perdikides T, Melas N (2006). Predicting the risk of rupture of abdominal aortic aneurysms by utilizing various geometrical parameters: revisiting the diameter criterion. Angiology.

